# Understanding the burden of illness of excessive daytime sleepiness associated with obstructive sleep apnea: a qualitative study

**DOI:** 10.1186/s12955-020-01382-4

**Published:** 2020-05-07

**Authors:** Laura Tesler Waldman, Sairam Parthasarathy, Kathleen F. Villa, Morgan Bron, Shay Bujanover, Meryl Brod

**Affiliations:** 1 The Brod Group, 219 Julia Ave, Mill Valley, CA 94941 USA; 2 University of Arizona Health Sciences Center for Sleep and Circadian Sciences and Division of Pulmonary, Allergy, Critical Care & Sleep Medicine, University of Arizona, Tucson, AZ USA; 3 Jazz Pharmaceuticals, Palo Alto, CA USA

**Keywords:** Obstructive sleep apnea, Excessive daytime sleepiness, Health-related quality of life, OSA, Sleepiness, HRQOL, Qualitative research, Work productivity, Impact on daily living, Daily function

## Abstract

**Background:**

Obstructive sleep apnea (OSA) is associated with excessive daytime sleepiness (EDS), which may go undiagnosed and can significantly impair a patient’s health-related quality of life (HRQOL). This qualitative research examined timing and reasons patients sought medical care for their EDS and OSA symptoms, and the impact of EDS on HRQOL.

**Methods:**

Focus groups were conducted in 3 US cities with 42 participants currently experiencing EDS with OSA. Transcripts were coded and analyzed using an adapted grounded theory approach common to qualitative research.

**Results:**

Over three-fifths of study participants (*n* = 26, 62%) were currently using a positive airway pressure (PAP) or dental device; one-third (*n* = 14, 33%) had previously used a positive airway pressure (PAP) or dental device, and the remainder had either used another treatment (n = 1, 2%) or were treatment naïve (n = 1, 2%). Twenty-two participants (52%) reported experiencing OSA symptoms for ≥1 year, with an average duration of 11.4 (median 8.0, range 1–37) years before seeking medical attention. Several (*n* = 7, 32%) considered their symptoms to be “normal,” rather than signaling a serious medical condition. Thirty participants (71%) discussed their reasons for ultimately seeking medical attention, which included: input from spouse/partner, another family member, or friend (*n* = 20, 67%); their own concern about particular symptoms (n = 7, 23%); and/or falling asleep while driving (*n* = 5, 17%). For all 42 participants, HRQOL domains impacted by EDS included: physical health and functioning (*n* = 40, 95%); work productivity (*n* = 38, 90%); daily life functioning (*n* = 39, 93%); cognition (n = 38, 90%); social life/relationships (*n* = 37, 88%); and emotions (*n* = 30, 71%).

**Conclusions:**

Findings suggest that patients may be unaware that their symptoms could indicate OSA requiring evaluation and treatment. Even following diagnosis, EDS associated with OSA can continue to substantially affect HRQOL and daily functioning. Further research is needed to address diagnostic delays and unmet treatment needs for patients with EDS associated with OSA.

## Background

Obstructive sleep apnea (OSA) is a condition in which the upper airway is obstructed during sleep, producing arousals from sleep and excessive daytime somnolence. OSA is associated with cardiovascular disease and other comorbidities, and may result in cognitive and performance deficits, such as difficulties with cognitive processing, sustaining attention, memory, and executive function [1–4]. One of the major presenting symptoms of OSA is excessive daytime sleepiness (EDS), which has the potential to significantly impair a patient’s health-related quality of life (HRQOL), including daily functioning [5–7], physical health and functioning [8, 9], emotional well-being [6, 9], social life [10], and cognition [11]. Patients with OSA have a 2–3 times overall increased risk for experiencing a driving accident [12, 13]. Emerging research examining work-related impacts of EDS associated with OSA in a broader range of occupations indicates that work productivity, absenteeism, presenteeism, risk of occupational injury, changes in job duties, opportunities for job promotions, and other aspects may also be negatively affected [14–18].

A number of studies provide evidence that both EDS and OSA are often undiagnosed [3, 19–23]; even among those patients with an OSA diagnosis, EDS may go undiagnosed and untreated [24]. Currently available treatments for OSA, such as continuous positive airway pressure (CPAP) and oral appliances, are frequently underused or rejected by patients due to discomfort, insurance, perceptions of efficacy, and other issues [25–28]. Even among those adherent to CPAP, EDS is estimated to persist for 12–65% of the population [29–32].

Information on the experience of EDS with OSA from a patient perspective is limited. Qualitative research methods, which ask open-ended questions of study participants and allow for the interviewer to probe further or generate new questions based on participant responses, are especially well-suited for eliciting patient perspectives [33]. Previous qualitative work conducted in OSA includes examination of patient treatment experiences and adherence [25, 28, 34, 35], OSA awareness and perceptions among taxi drivers [36], physician OSA management challenges [37], gender-based perspectives and experiences [38, 39], patient-partner relationships and experiences [38, 40, 41], and health care seeking [39, 42]. Scant research has used qualitative methodology to specifically examine EDS associated with OSA [10], and no currently published research has comprehensively assessed the impacts of this condition primarily on HRQOL and patient functioning using qualitative methods. The purpose of this study was to examine, using a patient-centric approach, the burden of illness experienced by patients with EDS associated with OSA and the timing and reasons patients sought medical care for their EDS and OSA symptoms. Such information can inform the selection of outcome measures that are important to patients and impact their daily functioning, and guide future research to optimize treatment strategies.

## Methods

Six semi-structured focus groups were conducted with people who were currently experiencing EDS associated with OSA in 3 US cities: New York City, Dallas, and Los Angeles. Recruitment was conducted via a professional market research organization, utilizing their proprietary databases of potential study participants consisting of people with a range of health conditions who had previously indicated an interest in participating in a research study after having been identified through multiple recruitment strategies, including advocacy organizations, patient support groups, online marketing, live events and community activity. A purposive sampling approach was used to select among the participants meeting the eligibility criteria in order to ensure variation and balance in the sample. Recruitment targets were therefore set for treatment status and demographic variables including gender, age, race/ethnicity, body mass index, income level and employment status. Among these targets, efforts to obtain equal proportions by gender, treatment status (current vs. previous device users), and a range of age groups were prioritized. Focus groups were conducted separately by gender. Participants completed a demographic and health form prior to the start of the focus group.

Inclusion criteria were: able to read, write, and speak English; be between 18 and 75 years of age; report a physician diagnosis of OSA; be currently treating or have previously treated their OSA with a physician-prescribed device (e.g., CPAP, bilevel positive airway pressure [BiPAP], oral appliance) at least once, or have undergone a procedure, e.g., surgery, to treat their OSA; have a usual sleep time of ≥6 h per night; and have a score of ≥11 on the Epworth Sleepiness Scale (ESS). Exclusion criteria were: having a cognitive impairment or any other medical condition that would impact their ability to participate in a focus group about their experience with EDS associated with OSA; a history or presence of bipolar disorder, bipolar related disorders, schizophrenia, or other psychotic disorders; having any other clinically relevant medical, behavioral, or psychiatric disorder other than OSA that is associated with EDS; having a nicotine dependence that has an effect on sleep, e.g., routinely awakening at night to smoke; or current, past (within past 2 years), or seeking treatment for a substance-related disorder or having a current or past (within past 2 years) diagnosis of a substance use disorder.

All focus groups were conducted in person at focus group facilities and moderated in English by professionals experienced in focus group leadership (co-authors L Waldman and M Brod). A focus group discussion guide was developed based on a literature review and designed to elicit participants’ experiences with EDS across several dimensions of HRQOL. The guide was semi-structured, consisting of a core set of open-ended questions concerning the symptom, diagnosis, and treatment experiences of participants, and the perceived impacts of EDS associated with OSA on physical health and functioning, daily life, work productivity, social and emotional well-being, and cognition. In keeping with qualitative research methodology to avoid biasing participants’ responses, the flow of the questions was funnel-shaped [43], starting with broad general questions about participants’ experiences with EDS associated with OSA before moving to domain-specific (i.e., each HRQOL category) questions, and then starting each domain-based section of the guide with general questions before asking about specific issues within the domain. In a semi-structured interview, questions in the interview guide do not need to be asked verbatim or sequentially (apart from starting with general questions prior to specific ones). Impromptu follow-up questions may also be asked based on participant responses to the core set of questions in the guide or other relevant topics introduced by participants during the focus group discussion. This permitted the focus groups to be conducted in an open-ended, conversational style, following the general scope of the guide, but also responding to participants’ thoughts. Efforts were made throughout each group to solicit responses from all participants on each topic to ensure that the data was not limited to the views and experiences of the most vocal members.

The focus groups lasted approximately 2 h and were audio recorded and transcribed. Participant names were replaced with unique ID numbers in the transcripts prior to analysis. This study followed a protocol that was approved by a central independent review board (IRB), the Copernicus Group IRB, located in Research Triangle Park, North Carolina. Informed consent was obtained from all participants. Participants received an honorarium for their participation commensurate with their time and effort.

Data were qualitatively analyzed through an adapted grounded theory approach, entailing developing and refining a theory based on concepts derived during the research process [44]. Transcripts were analyzed for content using Dedoose (www.dedoose.com), a qualitative analysis software program. A preliminary code list was created based on the discussion guide’s sensitizing (initial, general) concepts [45] and subsequently revised based on concepts derived from the focus groups during the coding process. Each transcript was initially read, then coded, and reviewed multiple times to ensure accuracy and consistency. Transcripts were coded in chronological order, and emerging concepts were added to the coding scheme as they arose. Earlier transcripts were then re-evaluated for the new concepts. As part of this iterative process, the coding scheme was continuously refined, including merging codes determined to be redundant and modifying or deleting codes as more nuanced understandings of concepts and their perceived relevance and importance to study participants emerged. The original sensitizing concepts selected for exploration during the focus groups were all found to be meaningful for participants, but were elaborated and refined over the course of the analysis.

Conceptual saturation (the point when no new important or meaningful concepts emerge) [44] was assessed for the 6 focus groups in the order in which they occurred. After the third focus group, 75% of concepts had been discussed; by the fifth focus group, 94% of concepts were covered and all meaningful concepts had emerged, confirming that the study sample size was sufficient. The relevance of the HRQOL domains explored during the focus groups were also confirmed during the saturation assessment.

Based on the study findings, a conceptual model was developed that captures the burden of illness for adult patients who experience EDS with OSA. For an impact to be included in the model, it had to be reported as relevant by ≥20% of the study sample. The model also includes potential modifiers that may impact individual experiences with the condition, based on a review of the literature.

## Results

### Sample description

Forty-two individuals participated in the focus groups. Self-reported demographic and health characteristics of the sample are shown in Table 1. Over half of the participants (*n* = 22, 52%) were male, and the average age was 51.4 (range 31–75) years. Nearly three-quarters (*n* = 31, 74%) were working full-time or part-time in a variety of positions across 13 occupational sectors, including health care, social work, education, finance, security guard services, and administrative.

**Table 1:** Participant Demographic and Health Characteristics

Demographic and Health Characteristics	Total (*n* = 42)
Gender, n (%)	
Female	20 (48)
Male	22 (52)
Age, years, mean, median (range)	51.4, 49.5 (31–75)
Race/Ethnicity, n (%)	
Asian American	4 (10)
Black/African American	4 (10)
Latino/Hispanic	5 (12)
Mixed race/ethnicity	1 (2)
White/Caucasian	28 (67)
Combined Household Annual Income, n (%)	
< $20,000	1 (2)
$20,001–$40,000	5 (12)
$40,001–$60,000	7 (17)
$60,001–$80,000	4 (10)
$80,001–$100,000	9 (21)
> $100,000	15 (36)
Decline to answer	1 (2)
Age at OSA Diagnosis, years, mean, median (range)	43.5, 44.0 (15–68)
Weight Status, n (%)	
Underweight/normal weight (BMI ≤24.9 kg/m^2^)	7 (17)
Overweight (BMI 25.0–29.9 kg/m^2^)	14 (33)
Obesity Class I (BMI 30.0–34.9 kg/m^2^)	4 (10)
Obesity Class II (BMI 35.0–39.9 kg/m^2^)	8 (19)
Obesity Class III (BMI ≥40 kg/m^2^)	9 (21)
Number of Comorbidities, mean, median (range)	2.0, 2.0 (0–6)
Number of Hours of Sleep Received per Night, mean, median (range)	6.7, 7 (6–8)
Average Hours of Sleep Received per Night, n (%)	
< 7 h	19 (45)
≥7 h	23 (55)
Nights per Week Wake up in Middle of Night or Too Early, n (%)	
0–1 night	4 (10)
2–3 nights	7 (17)
4–5 nights	16 (38)
6–7 nights	15 (36)
Number of Times Wake Up per Night, n (%)	
1 time	9 (21)
2 times	14 (33)
3 times	10 (24)
≥ 4 times	9 (21)
OSA Treatment Status, n (%)	
Currently uses CPAP/BiPAP or mandibular device	26 (62)
Previously used CPAP/BiPAP or mandibular device	14 (33)
Treatment-naïve or other treatment	2 (5)

*OSA* obstructive sleep apnea, *BMI* body mass index, *CPAP* continuous positive airway pressure; *BiPAP* bilevel positive airway pressure

Participants reported sleeping an average of 6.74 h per night (median 7, range 6–8). Over half (*n* = 24, 57%) reported waking up in the middle of the night or too early ≥5 nights per week. Over three-fifths (*n* = 26, 62%) were currently using a positive airway pressure (PAP) or dental device. Based on self-reported frequency of usage (days per week and hours per night), nearly two-thirds of current PAP users (*n* = 13/20, 65%) were adherent based on Centers for Medicare and Medicaid Services (CMS) criteria [46].

### Timing of diagnosis and reasons for seeking medical care

The mean age of diagnosis was 43.5 (range 15–68) years. Twenty-two participants (52%) reported experiencing OSA symptoms for ≥1 year, with an average of 11.4 years (median 8.0 years, range 1–37 years), before seeking medical attention. During this period, these participants reported snoring (*n* = 20, 91%) and EDS (n = 13, 59%) as the most frequently experienced symptoms. Several (*n* = 7, 32%) had considered their symptoms to be “normal” rather than a sign of a serious medical condition:

*I figured I’m just tired. I’m just always going to be tired. This is just kind of how life is. (Female, age 39)*

Thirty participants (71%) discussed their reasons for ultimately seeking medical attention for their OSA symptoms. Among these participants, the primary reasons were due to input from spouse/partner, another family member, or friend (*n* = 20, 67%), the participant’s own concern about particular symptoms (n = 7, 23%), and/or falling asleep while driving (*n* = 5, 17%). Small numbers also reported seeking medical attention due to having a comorbidity (n = 2, 7%), falling asleep at work (n = 2, 7%), having a car accident due to EDS (*n* = 1, 3%), being required by an employer (n = 1, 3%), and seeing a sleep study advertisement (n = 1, 3%).

### Descriptions of EDS

When asked to generally describe what it was like to experience EDS, participants most frequently reported feeling tired or tiredness (*n* = 37, 88%), experiencing “brain fog” or other terms for “feeling out of it” (*n* = 21, 50%), feeling sleepy or sleepiness (*n* = 18, 43%), feeling exhausted (*n* = 11, 26%), feeling like one never gets enough sleep (n = 11, 26%), and experiencing fatigue (*n* = 8, 19%):

*You obviously don’t have the level of high acuity that I think all of us would have normally. It’s a fog. You’re in a fog. (Male, age 45)*

### Impacts of EDS on HRQOL

The vast majority of study participants reported experiencing impacts from their EDS symptoms across all 6 HRQOL domains covered in the discussion guide: physical health and functioning (*n* = 40, 95%); work productivity (*n* = 38, 90%); daily life functioning (*n* = 39, 93%); cognition (n = 38, 90%); social life/relationships (*n* = 37, 88%); and emotions (*n* = 30, 71%).

### Impacts of EDS on physical health and functioning

**Fig. 1:**
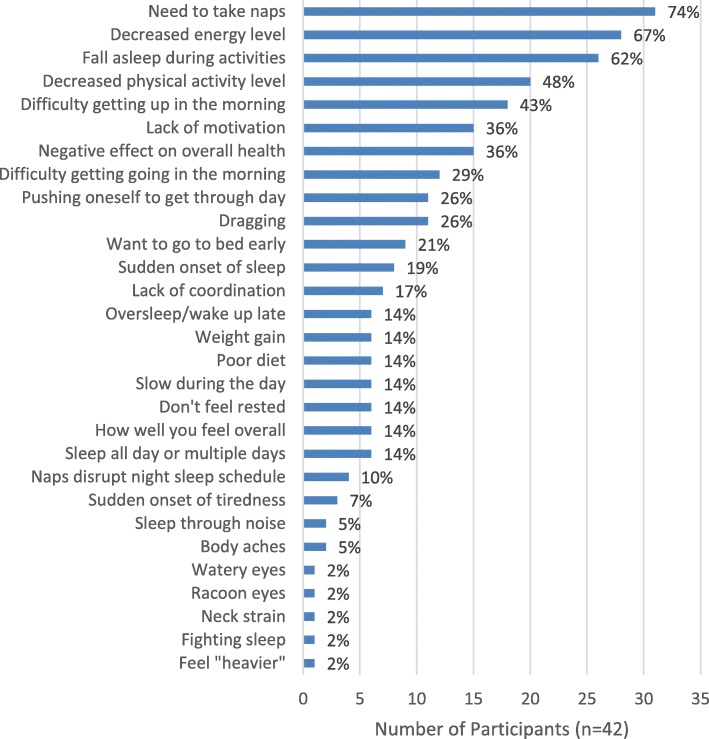
Impacts of EDS on Physical Health and Functioning*. EDS = excessive daytime sleepiness. *Participants were allowed to report > 1 impact

Nearly the entire sample (n = 40, 95%) reported that EDS affected their physical health and functioning (Fig. 1). Nearly three-quarters of the study sample (*n* = 31, 74%) reported taking naps due to EDS:

*In the afternoons, I have to take a nap usually for 2 hours. (Female, age 39)*

Over two-thirds of participants (*n* = 28, 67%) reported that EDS adversely affected their energy levels. Nearly half (*n* = 20, 47%) reported that their physical activity level had decreased due to EDS:

*Who wants to go to work out or ride a bike or play basketball … go shopping or anything when you’re feeling so tired? (Male, age 55)*

Sixteen participants (38%) believed EDS had adversely affected their overall health, either in general or by exacerbating comorbidities. Some participants also felt that EDS indirectly harmed their health by contributing to the development of unhealthy lifestyle habits:

*I’ve gained a little bit more weight because of lack of activity. At times when I would go to the gym, I just feel like relaxing and watching TV. (Male, age 37)*

### Impacts of EDS on work

**Fig. 2:**
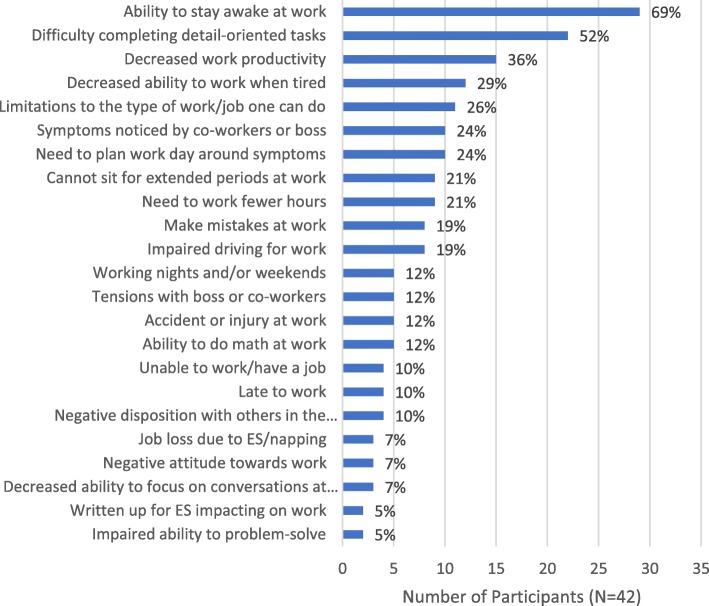
Impacts of EDS on Work*. EDS = excessive daytime sleepiness. *Participants were allowed to report > 1 impact

Ninety percent of participants reported either currently (*n* = 29, 69%) or previously (*n* = 9, 21%) experiencing EDS-related work impacts (Fig. 2). Nearly 70% (n = 29, 69%) reported that EDS affected their ability to stay awake at work, often resulting in their needing to take naps:

*If I get sleepy enough … I’ll get up and shut my door just to protect myself … Sometimes it’s 10 minutes. There’s been occasions where it’s like 40. (Male, age 61)*

Approximately half of participants (*n* = 22, 52%) reported that EDS impacted their ability to carry out detail-oriented tasks at work*.* Just over a third (*n* = 15, 36%) reported that EDS affected their productivity at work, both in general and with respect to how long it took them to get things done, as well as impacting their ability to meet deadlines:

*There’s definitely times where I feel like I could be more productive. (Male, age 48)*

Nineteen participants (45%) identified work-related EDS symptom triggers, including attending meetings/presentations, sitting for extended periods, and computer work:

*We just had a webinar and it was a room full of people … The person’s voice is kind of monotone … I dozed off. (Female, age 41)*

Strategies for managing EDS in the workplace included consuming caffeine, taking naps, planning the workday around symptoms, and taking breaks for light physical activity. Nevertheless, 10 participants (24%) reported that symptoms had been noticed by their boss or co-workers; 5 (12%) subsequently experienced disciplinary action, including employment termination (*n* = 3, 7%):

*After lunch … my call logs would go from going full pace to 2 or 3 calls after twelve o’clock … And that’s where I got fired. (Female, age 31)*

Eleven participants (26%) reported that EDS affected the type of work they could do or choice of occupation:

*I was working at an auto parts company … And I had nodded out into my machine before and that can’t happen. That’s dangerous … Now I’m taking care of elderly people. (Female, age 45)*

### Impacts of EDS on daily life

**Fig. 3:**
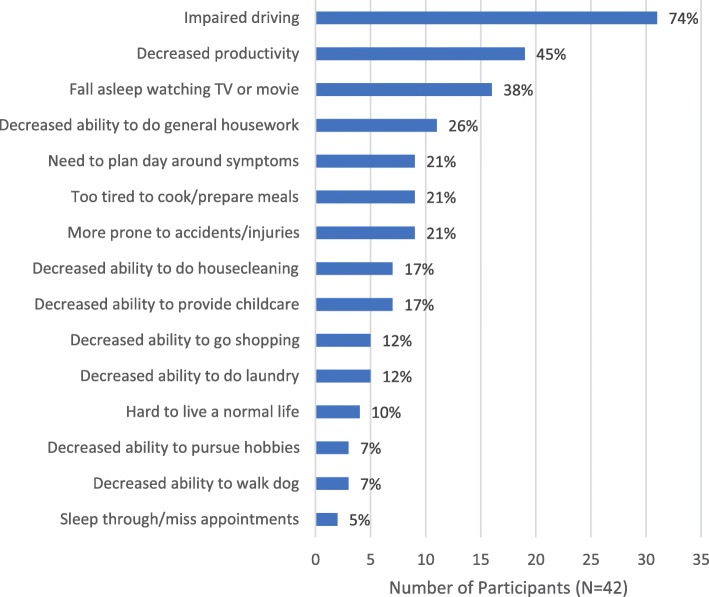
Impacts of EDS on Daily Life*. EDS = excessive daytime sleepiness. *Participants were allowed to report > 1 impact

Over 90% of participants (*n* = 39, 93%) reported that EDS had affected their daily lives outside of the workplace (Fig. 3). Nearly three-quarters (*n* = 31, 74%) reported that EDS affected their driving:

*You just close your eyes for a second … and a second turns into a couple of minutes … And the light changes. People are beeping behind you. I’m lucky if I don’t let go of the brake. (Male, age 55)*

Six participants (14%) described themselves as driving on “auto-pilot” or a similar state whereby their eyes remained open, but they believe they were sleeping*.* Four participants reported having been in car accidents due to their EDS, and one of them stopped driving altogether as a result.

Nineteen participants (45%) reported that EDS affected their overall productivity in daily life:

*I don’t do anything anymore. You know, I’m not that old, but I have no desire to get out and do anything. I just stay at home and watch TV. (Male, age 58)*

Eleven participants (26%) reported that EDS interfered with their ability to do general housework or chores:

*I think, ‘I’m too tired right now, I’ll do it later.’ Later has yet to come and that’s been several years. (Female, age 63)*

Seven participants (17%) reported that EDS impeded their caregiving ability:

*I want to be able to do homework and be in a bright, cheery, happy mood like, ‘Hey, what are we learning today?’ And instead it’s like, ‘Ok, come on; let’s do your homework.’ … I want to be there with them, but my body is exhausted. (Female, age 38)*

As in the workplace, several participants (*n* = 9, 21%) reported planning their day around their EDS symptoms, to maximize their productivity or to be able to go out in the evening:

*I have to adjust my schedule accordingly so maybe try to nap during the day before we have to go at night or something like that. (Male, age 32)*

### Emotional impacts of EDS

**Fig. 4:**
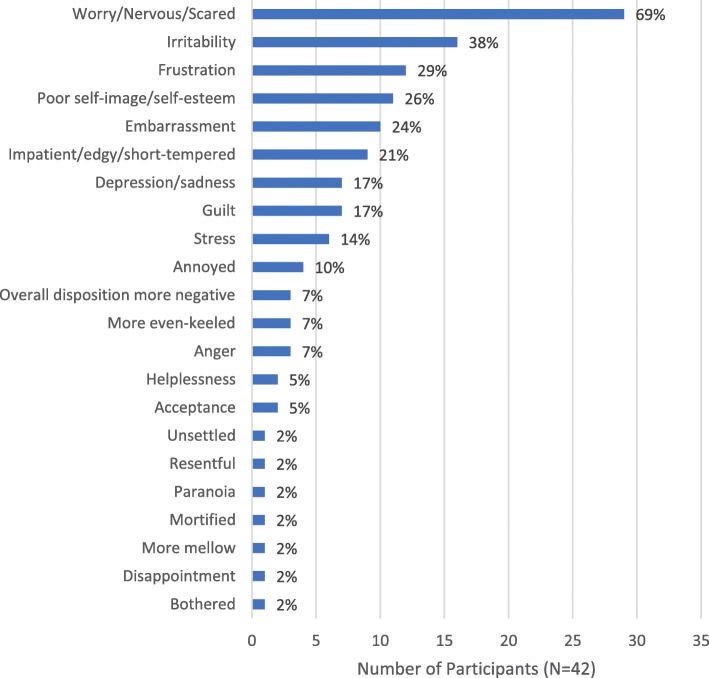
Emotional Impacts of EDS*. EDS = excessive daytime sleepiness. *Participants were allowed to report > 1 impact

Over 70% of participants (*n* = 30, 71%) reported experiencing emotional impacts from EDS (Fig. 4), especially worry (*n* = 29, 69%), most frequently about the risk of having a car accident (*n* = 18, 43%):

*That’s an actual conscious worry that I have quite frequently … I’m always struggling to keep my eyes open when I’m driving a long distance or even when I’m driving a moderate distance. (Male, age 54)*

Four participants (10%) worried about memory loss they attributed to EDS:

*My mental alertness or impact on my memory can have an impact on my livelihood. (Male, age 45)*

Nearly two-fifths of participants (*n* = 16, 38%) attributed their irritability, crankiness, moodiness, or grumpiness to their condition. Eleven (26%) reported that their EDS had negatively affected their self-image or self-esteem, especially when they compared their levels of energy and productivity with others, or felt like they were not accomplishing daily tasks or life goals. Approximately one-quarter (*n* = 10, 24%) reported experiencing embarrassment due to their EDS, especially when they fell asleep in front of others or during activities with others:

*I’m embarrassed to fall asleep, because I know it’s bound to happen, especially if I’m in public … (Female, age 41)*

### Social impacts of EDS

**Fig. 5:**
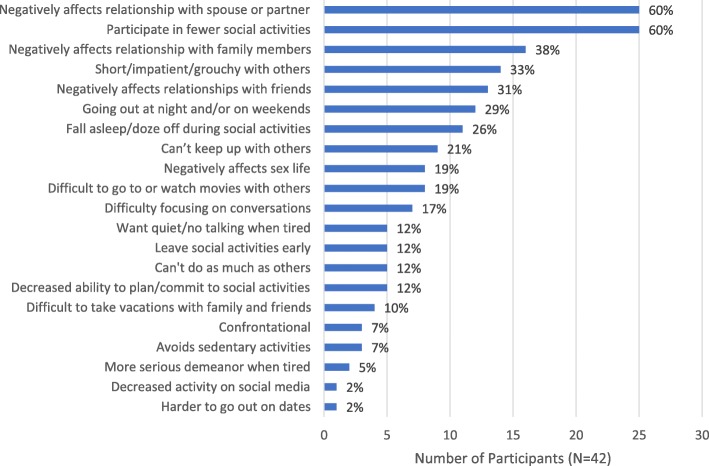
Social Impacts of EDS. EDS = excessive daytime sleepiness. *Participants were allowed to report > 1 impact

Nearly 90% of participants (*n* = 37, 88%) reported that EDS affected their social lives and relationships (Fig. 5), especially relationships with a current or former spouse/partner (*n* = 25, 60%). Specific sources of tension included a spouse/partner feeling like they did not get to spend enough quality time with the participant due to their sleepiness and sleep schedule, and resentment that the participant was not as productive as the spouse/partner felt they should be:

*My husband gets upset with me because he gets up in the morning, he gets things done … if I got up earlier, I’d still feel sleepy. By 9 o’clock, I’d be wanting to take a nap. (Female, age 63)*

Many participants also reported that relationships with family (*n* = 16, 38%) and/or friends (*n* = 13, 31%) had been affected. One-third (*n* = 14, 33%) reported being short, impatient, or grouchy with others due to their EDS:

*My wife has complained that I have become either nasty, quiet, or angry … A lot of that has to do with the fact that I’m just tired. (Male, age 75)*

Just over one-quarter of participants (*n* = 11, 26%) reported falling asleep or dozing off during social activities, and 60% (*n* = 25) reported participating in fewer social activities due to their EDS. Correspondingly, just over one-fifth reported being unable to “keep up” with others:

*My sister, we’re only a year apart … she’s going to have done ten things before I get up and have breakfast. (Female, age 49)*

### Cognitive impacts of EDS

**Fig. 6:**
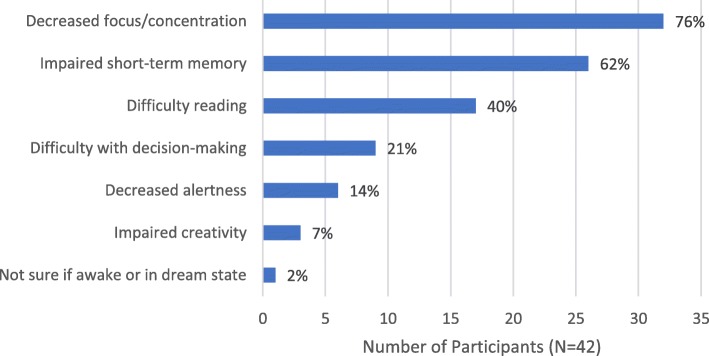
Cognitive Impacts of EDS. EDS = excessive daytime sleepiness. *Participants were allowed to report > 1 impact

Ninety percent of participants (*n* = 38) reported that EDS had affected their cognition (Fig. 6), disrupting both their work and personal lives. Three-quarters (*n* = 32, 76%) reported that EDS impaired their ability to focus or concentrate*.* Over three-fifths (*n* = 26, 62%) reported that EDS adversely affected their short-term memory. Two-fifths (*n* = 17) reported having difficulty reading, finding it difficult to concentrate on the text or falling asleep*.* One-fifth (*n* = 9, 21%) reported that EDS impacted their decision-making ability:

*I sometimes tend to analyze more, even for simple things sometimes, just to make sure that I’m thinking in the right state of mind. (Male, age 37)*

### Conceptual model

**Fig. 7:**
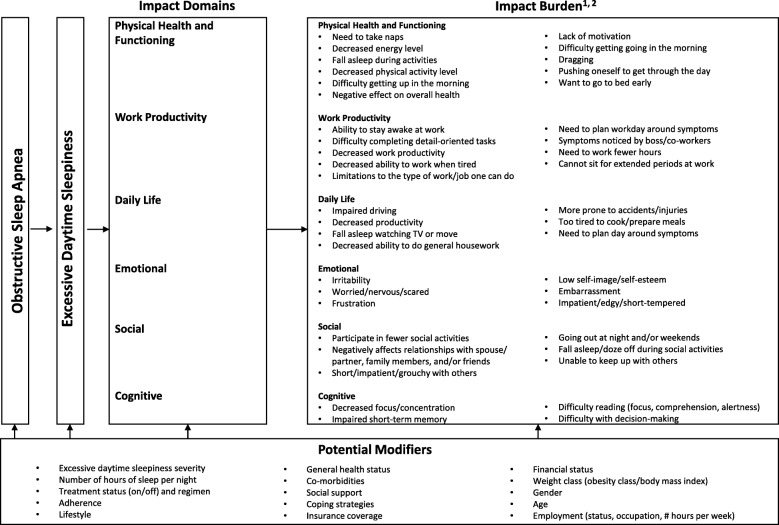
Conceptual Model of the Burden of Illness of EDS Associated with OSA^*,†^. EDS = excessive daytime sleepiness; OSA = obstructive sleep apnea; BMI = body mass index. *Model includes all impact burdens reported by ≥20% of study participants. ^†^Participants were allowed to report > 1 impact for each domain

Based on the study findings, a conceptual model was developed that captures the burden of illness for adult patients who experience EDS with OSA (Fig. 7). The domains with the greatest number of impacts that affect ≥20% of the study sample were physical health and functioning and work productivity. Several potential modifiers that may impact individual experiences with the condition were also incorporated into the model, including EDS severity, amount of sleep received per night, treatment status/regimen, age and comorbidities.

The conceptual model further illustrates that modifiers may affect not only the impacts experienced by patients but also the condition itself. For example, lifestyle factors that contribute to an insufficient amount of sleep or co-morbidities, such as depression, may exacerbate or even be the underlying cause of EDS. Similarly, lifestyle factors, adherence, treatment status and other modifiers may impact the severity of one’s OSA, which may in turn affect the severity of one’s EDS and its associated impacts.

## Discussion

Study findings suggest that EDS associated with OSA puts a tremendous physical and mental health burden on adult patients, with respect to the range of impacts, including functioning, cognition, safety, emotions, and relationships. Over two-thirds of study participants attributed EDS to impaired functioning across multiple HRQOL domains, and nearly three-quarters cited driving issues, which could have an effect on public health and safety. Certain impacts, such as driving and cognition, have been described in several previous studies; however, to the best of our knowledge, this is the first study to use qualitative methodology to comprehensively examine the overall burden experienced by patients with EDS associated with OSA on HRQOL and patient functioning.

Also to the best of our knowledge, no previous burden of illness model for this condition has been published. This study further expands our understanding about the specific burden of EDS associated with OSA, and the model, while driven by the study data, pools together and confirms many of the concepts included in existing OSA-specific instruments. It should be noted that not all OSA-specific instruments have been developed based on patient input. Among those that have included patient input, the development histories available in the public domain do not provide the details on the participant endorsement rates for each impact identified as has been done for the current study, nor do they include participant quotes which offer context and clarity for the intent of each item. This model and the in-depth description of the data underlying its development may thus serve as a useful resource for other researchers as well as clinicians.

In addition, many existing OSA-specific instruments include items covering impacts potentially related to multiple OSA symptoms, without a means of determining whether and to what extent a particular symptom contributes to a particular impact. The ability to directly identify and measure the burden of EDS in particular is important since, as previously noted, some patients continue to be impacted by EDS even when using a PAP device. This persistence suggests that there may be a need for treatment interventions specifically targeting EDS. Increased understanding of the impact burden of EDS associated with OSA in particular, as opposed to EDS more broadly, may further provide insights into targeted treatment interventions appropriate for this population.

The potential for modifiers to directly affect not only the impacts of this condition but also EDS in particular and OSA more broadly suggests that the relationships depicted in the model are complex. Moreover, not all modifiers included in the model may be relevant for each individual patient, and there may be additional modifiers which have not been identified through this research or the literature. Future research with a quantitative sample would be useful for further elucidating the potential connections between and among the illness, impacts and modifiers of EDS associated with OSA.

Many impacts described in this research have affected not only the participants but their spouses/partners, family members, and friends; three-fifths reported that EDS had a negative impact on their relationship with their spouse/partner and resulted in their participating in fewer social activities. While little research has been conducted regarding the impact of EDS associated with OSA on partner relationships and social activities, these findings are consistent with previous studies in which a reduced social life, marital issues, and negative impacts on partner intimacy have been attributed to this condition [10, 47, 48]. In the present study, marital tensions appeared to go beyond problems of sexual intimacy to encompass concerns about reductions in spousal productivity and quality time available for couples to spend together, along with patient guilt for falling short in these areas.

The number and type of work impacts reported by high proportions of study participants further suggest that EDS with OSA may affect work productivity in a broad range of occupations, in line with emergent findings from other studies [15–17]. The ability to stay awake and to complete detail-oriented tasks at work were especially challenging for participants in the current study. Future research should quantitatively characterize the impacts of EDS on work productivity and examine how treatments and interventions might address them.

Study findings also suggest that patients may be unaware that their symptoms could indicate OSA requiring evaluation and treatment. Among these patients, there was an average of over 11 years between symptom onset and seeking medical attention. This delay is troubling, but perhaps unsurprising given evidence that OSA is underdiagnosed [3, 19, 20]. Notably, several participants did not consider their symptoms to be serious enough to warrant medical attention, and half of the study sample attributed a spouse/partner, family member, or friend as the primary motivating factor for ultimately seeking care. Similar findings have been reported by Zarhin in her study of health care-seeking for OSA in an Israeli population [39]. Even since receiving a diagnosis, participants across treatment regimens, including those with high CPAP adherence, continue to suffer from EDS. This finding, which has also been documented elsewhere [11], suggests that current OSA treatments may not adequately address EDS, and patient care may be suboptimal for this population.

### Study limitations

As a qualitative, exploratory study that included only 42 adult patient participants on multiple treatment regimens, conclusions drawn from this dataset are suggestive and not definitive. However, for the purpose of this study, the respondent sample is more than adequate for exploring the experiences and burdens related to EDS associated with OSA.

This study used a purposive sampling approach, which incurs a risk of sampling bias. However, the research team decided to use this approach out of concern for maximizing the variation of treatment and demographic characteristics in the sample, having determined based on prior experience that using a consecutive sampling approach for a small study sample would likely have resulted in under-representation for one or more key variables.

Due to the inclusion criterion requiring that participants receive an average of ≥6 h of sleep per night, the study sample was relatively homogeneous with respect to the amount of sleep participants received, which precludes an examination of the differences in the burden of illness based on this attribute. In addition, an ESS score of ≥11 was required for participation in these focus groups, and in the general OSA population, the impacts of EDS might be more variable. This inclusion criterion also precluded the opportunity to examine the potential impacts of PAP devices and other treatments on alleviating EDS in patients with OSA. A survey study with a more representative sample of the general population of people who experience EDS with OSA would need to be undertaken to assess how impacts may vary based on amount of sleep, treatment regimen, and treatment adherence. Such research would also be appropriate for exploring potential variations identified in other studies, including gender [49, 50].

## Conclusions

The study findings suggest that patients may be unaware that symptoms they experience could indicate OSA that requires evaluation and treatment, leading to delays in diagnosis. Even following diagnosis, EDS associated with OSA can continue to place a substantial burden on patients with respect to both the range and seriousness of impacts, including impairments in functioning, cognition, work, safety, and relationships. A majority of participants in the study sample reported the need to take naps, decreased energy level, decreased ability to focus or concentrate, decreased ability to stay awake or complete detail-oriented tasks at work, impaired driving, and tensions in relationships with spouses/partners, family members, and friends. Further research is needed to address diagnostic delays and unmet treatment needs for patients with EDS associated with OSA, as well as additional patient education on the disease and its implications. A quantitative survey study would be appropriate to further explore potential variations among subgroups and the overall impact of EDS with OSA.

## Data Availability

The datasets used and/or analyzed during the current study are available from the corresponding author on reasonable request.

## Abbreviations

OSA: Obstructive sleep apnea
EDS: Excessive daytime sleepiness
HRQOL: Health-related quality of life
CPAP: Continuous positive airway pressure
BiPAP: Bilevel positive airway pressure
ESS: Epworth sleepiness scale
IRB: Independent review board
PAP: Positive airway pressure
CMS: Centers for Medicare and Medicaid Services
